# Toward a reliable detection of arachnophobia: subjective, behavioral, and neurophysiological measures of fear response

**DOI:** 10.3389/fpsyt.2023.1196785

**Published:** 2023-06-08

**Authors:** Eva Landová, Silvie Rádlová, Anna Pidnebesna, David Tomeček, Markéta Janovcová, Šárka Peléšková, Kristýna Sedláčková, Iveta Štolhoferová, Jakub Polák, Jaroslav Hlinka, Daniel Frynta

**Affiliations:** ^1^Department of Zoology, Faculty of Science, Charles University, Prague, Czechia; ^2^National Institute of Mental Health, Klecany, Czechia; ^3^Institute of Computer Science of the Czech Academy of Sciences, Prague, Czechia; ^4^Department of Economy and Management, Ambis University, Prague, Czechia

**Keywords:** arachnophobia, fear, fMRI, snakes, spiders, behavioral approach test

## Abstract

**Introduction:**

The administration of questionnaires presents an easy way of obtaining important knowledge about phobic patients. However, it is not well known how these subjective measurements correspond to the patient’s objective condition. Our study aimed to compare scores on questionnaires and image evaluation to the objective measurements of the behavioral approach test (BAT) and the neurophysiological effect of spiders extracted from fMRI measurements. The objective was to explore how reliably subjective statements about spiders and physiological and behavioral parameters discriminate between phobics and non-phobics, and what are the best predictors of overall brain activation.

**Methods:**

Based on a clinical interview, 165 subjects were assigned to either a “phobic” or low-fear “control” group. Finally, 30 arachnophobic and 32 healthy control subjects (with low fear of spiders) participated in this study. They completed several questionnaires (SPQ, SNAQ, DS-R) and underwent a behavioral approach test (BAT) with a live tarantula. Then, they were measured in fMRI while watching blocks of pictures including spiders and snakes. Finally, the respondents rated all the visual stimuli according to perceived fear. We proposed the Spider Fear Index (SFI) as a value characterizing the level of spider fear, computed based on the fMRI measurements. We then treated this variable as the “neurophysiological effect of spiders” and examined its contribution to the respondents’ fear ratings of the stimuli seen during the fMRI using the redundancy analysis (RDA).

**Results:**

The results for fear ranks revealed that the SFI, SNAQ, DS-R, and SPQ scores had a significant effect, while BAT and SPQ scores loaded in the same direction of the first multivariate axis. The SFI was strongly correlated with both SPQ and BAT scores in the pooled sample of arachnophobic and healthy control subjects.

**Discussion:**

Both SPQ and BAT scores have a high informative value about the subject’s fear of spiders and together with subjective emotional evaluation of picture stimuli can be reliable predictors of spider phobia. These parameters provide easy and non-expensive but reliable measurement wherever more expensive devices such as magnetic resonance are not available. However, SFI still reflects individual variability within the phobic group, identifying individuals with higher brain activation, which may relate to more severe phobic reactions or other sources of fMRI signal variability.

## 1. Introduction

Specific phobia is defined as an excessive or unreasonable fear of a particular object, situation, or activity that poses little or no actual danger (1). It is a type of anxiety disorder that can have a significant impact on an individual’s life. According to the latest review by Eaton et al. (2), the median lifetime prevalence of specific phobias in the world is 7.2% and can range anywhere between 3 and 15%. This trend was confirmed by a survey conducted in 22 countries reporting a cross-national lifetime prevalence rate of 7.4 and 5.5% in a 12-month period (3). In general, phobias are significantly gender-dependent with women being affected as much as twice more often than men (4, 5).

Phobias can have a significant economic and social burden as well. Healthcare costs associated with anxiety disorders in primary care are estimated to be substantial (6). In the 1990s, the economic burden of anxiety disorders in the United States was estimated to be $42 billion (7). Furthermore, comorbid psychiatric disorders, such as depression, can increase the risk of relapse and hinder recovery in individuals with phobias, which can further increase the economic burden (8). Phobias can also have a significant social burden. For example, social phobia can lead to social isolation and loneliness, particularly in older women (9). This can further exacerbate the impact of the disorder on the individual’s life.

While the assessment of phobias is essential for accurate diagnosis and treatment planning, there can be challenges in conducting a thorough assessment. These challenges can arise from the nature of phobias themselves, as well as from limitations in assessment methods. One of the primary difficulties in assessing phobias is that individuals may avoid the feared object or situation, making it difficult to observe their response to it. This avoidance can also make it challenging to obtain accurate information about the onset and course of the phobia (10). In addition, individuals with phobias may be reluctant to disclose information about their symptoms due to embarrassment or fear of stigma (11).

Another challenge in assessing phobias is that self-report measures may not fully capture the complexity of the individual’s experience. For example, an individual may report a fear of spiders, but the self-report measure may not indicate the intensity or specific triggers of that fear. Furthermore, individuals may over-report or under-report symptoms based on their own biases or perceptions (12). Cognitive and physiological measures can also be challenging to use in the assessment of phobias. Cognitive measures may not be sensitive to the specific fears and beliefs of the individual, and physiological measures may be influenced by factors other than the phobia, such as general anxiety or physical or mental health (10, 13).

Finally, there are limitations to the existing assessment measures for phobias. While self-report measures such as the Fear Questionnaire are widely used, they may not be comprehensive enough to fully capture the range of phobias and associated symptoms. Behavioral observations may also be limited by the controlled environment of the clinic and may not fully reflect the individual’s response to the feared object or situation in the real world (14).

Many studies on snake or spider phobia base their results on respondents with high scores on the Snake Questionnaire [SNAQ: (15), in a Czech translation by Polák et al. (16)] or Spider Questionnaire [SPQ: (15), in a Czech translation by Polák et al. (17)]. It should be noted, however, that others have criticized that questionnaires alone may not represent a reliable measurement of specific phobias. For example, (18) argued against SNAQ due to its low construct and criterion validity as a significant number of people scoring high on the SNAQ were able to approach a caged snake. Klieger and Siejak (19) concluded that SNAQ can identify fearful individuals but is strongly biased by false positives because some of the items tap into disgust. Similarly, in the case of spider fear, various questionnaires were developed, but each showed only a moderate correlation with the behavior of the respondents: SPQ-C [SPQ modified for the use on children; (20)], SADS-C [Spider Anxiety and Disgust Screening for Children, (21)], FSQ [Fear of Spiders Questionnaire; (22)], or SPQ-15 [Reduced SPQ; (23, 24)].

There is an extensive line of evidence showing that specific phobias in general, and spider phobia in particular, are not only associated with intense fear but also have a significant disgust component (25). This was recently demonstrated in a study by Polák et al. (17) who reported a correlation coefficient of 0.40 between scores on measures of spider fear (SPQ) and disgust propensity (Disgust Scale – Revised, DS-R). Some authors suggest that spiders trigger contamination-based fear (26, 27). Gerdes et al. (28) showed that spiders are unique in eliciting significantly greater fear and disgust than any other arthropod [see also (29)]. A disease-avoidance model (30) hypothesizes that spider phobia develops from the convergence of the spiders’ disgusting properties and the subjective probability of involuntary physical contact with humans. Indeed, spiders are regarded as highly disgusting by healthy subjects and even more by people with arachnophobia (31), potentially due to their quirky ‘too-many-legs’ body plan. However, it proves difficult to disentangle fear and disgust elicited by spiders due to their tight correlation (32) and thus, in the following study, we decided to focus only on fear.

Moreover, as our own long-time experience in the field of animal phobias has shown, the variability among respondents that define themselves as highly fearful of spiders is very wide and an exact boundary to distinguish a phobic patient from a non-phobic respondent with high, yet non-clinical fear is very hard to define. Some respondents fear a wide variety of stimuli, including pictured spiders, or stress out from the knowledge of the animal being present in the room. Some others only fear living snakes and spiders and have no problems with pictures, but still may fall under the DSM-5’s definition of a phobic (1). Some respondents experience very high fear but can control it, while some are close to fainting face to their phobic object. Even people labeled as phobic have varying degrees of difficulty in everyday life. Questionnaires are thus good and practical tools to be used when searching for phobic or high-fear respondents in the population, but to precisely measure the severity of spider fear or phobia, specially designed tests (such as structured interviews combined with an anxiety anticipation test and physiological measurements) are needed.

Another reason why there are such hardships accompanying the measurement of fear may be that different components may contribute to the final experience and expression of fear (33). The dual-process models distinguish between implicit (automatic) and explicit (controlled) processes that modulate the avoidance behavior when experiencing fear and anxiety (34, 35). Klein et al. (36) investigated how the direct measurement that addressed explicit self-reports and subjective fear (using questionnaires) and the indirect measurement that addressed implicit aspects of fear processing (emotional Stroop task) impacted avoidance behavior in children. They showed that the emotional Stroop task and the self-reports both significantly correlated with the children’s behavior, but independently of each other. Similarly, in Effting et al. (37) found the moderating role of the working memory capacity of the implicit fear processing during BAT, confirming the dual-process hypothesis.

In another study, Huijding and de Jong (38) considered the results of BAT to be “relatively controllable,” measuring mostly explicit processing of the fearful behavior, and found a correlation with self-reported FSQ scores, while eye-blink startle response (implicit behavior) correlated with indirect measurements of fear (automatic associations). Similar results were found by Van Bockstaele et al. (39), who also treated BAT as a mostly explicit measurement of spider fear.

However, we can argue that the behavioral approach test does not measure “mostly” controllable behavior but is rather the result of both implicit and explicit processes. During BAT, the respondents are confronted with a real stimulus – either a real live spider or a dummy they believe is real. This knowledge alone can trigger an automatic, implicit response in respondents with high fear that can be modulated by explicit behavior to some extent – e.g., the respondent can push themselves beyond the limit because they feel pressured by the situation or can hold themselves back because they are being tested for spider fear and thus want to give a fearful response. Similarly, when respondents see visual stimuli in the form of spider pictures presented on a screen, they are confronted with the stimulus. The severity of the fear may be reduced because the stimulus is not real; but when scoring such stimuli for self-perceived fear, the implicit component should be still modulating the response, especially if the respondents are quick with their responses. As such, scoring of visual stimuli may prove to be a reliable and accurate technique to be used when measuring phobic fear.

In contrast, when measuring fear by self-report using questionnaires, the respondents are not confronted with a live spider or its visual representation. The respondents are only confronted with a mental representation of a spider, which, in some cases, may trigger an implicit reaction to some extent, especially in very strong phobic respondents that may faint or flee even when just hearing the word “spider.” However, in most subjects, the reaction will be composed mostly of the explicit component because they will need time to read (or hear) and understand the question and think about the truthful answer. The answer will always be slower and thus composed mostly of the explicit fear reaction.

In our study, we hypothesized that BAT and self-reported scores of fear from picture stimuli (fear scores) are composed of both implicit and explicit processes, and should be thus correlated with both self-reported, controlled measurement using a questionnaire, and measurement of an implicit component of spider fear. In such cases, both variables (BAT and fear scores) could be then considered for use as reliable measurements of spider fear for future experiments or clinical examination of phobic patients, because, unlike questionnaires alone, these measurements could also cover the implicit component of fear. A good assessment of implicit processing is the measurement of physiological reactions. We thus used data from functional magnetic resonance imaging (fMRI) to propose a “Spider Fear Index,” or shortly SFI. This index is based on the neural activation of spider-phobic respondents during the presentation of phobic stimuli inside the MRI tomograph and reflects the intensity of activation of areas that are active during the expression of phobic fear. Our aim was therefore to explore the relationship between different ways of measuring fear of spiders, with a particular focus on methods that combine implicit and explicit responses and their comparison with questionnaires.

We then concentrated on the parameters of SFI mirroring the intensity of neural activity of the brain while implicitly experiencing fear. Specifically, we asked whether the measurement SFI and the fear scores of picture rating can be a good predictor of spider phobia. In other words, we asked if the phobic respondents in our study (selected based on results from a structured psychological interview) could be discriminated into the “phobics” group (and separated from control respondents) only based on the SFI scores and picture fear scores alone. This question is very important because successful discrimination would show us that these measurements can, similarly to questionnaires, be reliably used to detect spider phobia. Additionally, if fear scores proved to be a good phobic predictor, it would further support using visual stimuli for the detection of spider phobia.

## 2. Materials and methods

### 2.1. Respondents’ recruitment

To recruit the respondents, we used a standardized Czech translation of the Spider Questionnaire [(15), translation: (17)], which had been developed using a back-translation procedure and then validated for the Czech population (*N* = 3,863). In the original sample, 10.3% of subjects reached the selected cut-off point for spider phobia (17). However, it has been noted that certain assessments of animal phobias tend to yield false positive results overestimating the actual rate of phobics (Kleiger and Siejak, 1997). Therefore, to avoid such a risk of identifying as spider phobics even healthy subjects, we have developed a structural interview based on the diagnostic criteria of spider phobia as defined in the Diagnostic and Statistical Manual of Mental Disorders [DSM-5: (1)]. To confirm a diagnosis of spider phobia, the interview was administered by a trained clinical psychologist only to respondents who reached score 22 (i.e., clinical threshold) on the SPQ (17) and those whom we had known from our previous study that suffered from an increased fear of spiders (29).

The structured interview was used together with the result of the SPQ questionnaire to classify the respondent as a phobic group. It focuses on the specific experience and history of the individual respondent; open-ended answers allow for a more detailed description of specific situations. The interview focuses on the extent to which the fear of spiders affects the respondent’s everyday life, as it is the negative impact on everyday functioning that is a necessary condition for the diagnosis of phobia, also according to diagnostic manuals [i.e., DSM-5: (1)]. It should also eliminate potential false positives that might occur (i.e., people with high scores that do not experience higher fear in their everyday life). The interview consisted of six questions assembled by a clinical psychologist and when five out of six questions implied high fear of spiders, the respondent was assigned to the “phobic” category. Example of a question: are you afraid of encountering a spider so much that you try to avoid possible encounters? If yes, give an example of such a situation.

In total, 134 clinical interviews were conducted with individuals suffering from an increased fear of spiders; a diagnosis of spider phobia was confirmed in 131 of them. Out of these, 30 phobics were randomly selected to participate in the following study (age 18–66 years, mean age 29.0 years, mean SPQ score 21.7, range 13–31), while the remaining subjects were recruited for other projects.

Furthermore, in our previous research (29), we identified people reporting no fear of spiders (answering “no” to the question “Do you fear spiders in general?”; *n* = 210). We randomly sampled respondents from this pool and invited them into this study as control subjects until we reached the sample size of 31. To confirm the low spider fear status of this group, each subject went through the same clinical interview as the phobics (age 19–58 years, mean 30.3 years, mean SPQ score 1.6, range 0–4). As a result, the control group scored 0 on all six questions of the clinical interview.

Only women were included in this study because, as mentioned above, they are more likely to have phobias and are more cooperative in research. The selected respondents from the phobic and control group took part in all the research phases, i.e., behavioral approach test (BAT), functional magnetic resonance (fMRI) measurements, and image evaluation.

### 2.2. Assessment battery

#### 2.2.1. Spider questionnaire (SPQ)

The spider questionnaire (SPQ) is a 31-item self-report scale to assess the verbal–cognitive component of spider fear originally developed by Klorman et al. (15) and recently translated into Czech by Polák et al. (17). Each item is a fearful or non-fearful statement related to spiders and is rated by the respondent as true or false. The instrument is scored by assigning a ‘1’ to each true response and ‘0’ to each false response, nine items are reversed-scored. A total score (ranging from 0 to 31) is calculated by summing all ‘true’ statements. Psychometric analyses have shown that the SPQ has a high internal consistency as estimated by Kuder–Richardson Formula 20 [e.g., 0.83–0.94: (15); or 0.81–0.89: (40)] or Cronbach’s alpha [*α* = 0.94: (17)], excellent test–retest reliability after 2 months [*r* = 0.93: (17)] or a year [e.g., *r* = 0.87: (40)], and satisfactory levels of validity as it can discriminate between people with arachnophobia and healthy controls (40, 41). There also exists its shortened 12-item version in Czech (24) and Hungarian (42).

#### 2.2.2. Snake Questionnaire (SNAQ)

The Snake Questionnaire (SNAQ) [developed by Klorman et al. (15); translated into Czech by Polák et al. (16)] is a 30-item self-report scale to assess the verbal-cognitive component of snake fear. Each item is a fearful or non-fearful statement related to snakes. Participants rate each item as true or false. The instrument is scored by assigning a “1” to each true response and “0” to each false response, nine items are reversed-scored. A total score (ranging from 0 to 30) calculated by summing all ‘true’ statements serves as a measure of the degree of phobic fear (43, 44). The SNAQ shows good internal consistency [0.78–0.90: (15) or 0.91: (16)] and excellent test–retest reliability [*r* = 0.84: (40); *r* = 0.94: (16)] and discriminates well between people with snake phobia and healthy controls (45). There also exists its shortened 12-item version in Czech (24) and Hungarian (42).

#### 2.2.3. Disgust Scale – Revised (DS-R)

The Disgust Scale – Revised (DS-R) [(46); modified by Olatunji et al. (47); translated to Czech by Polák et al. (48)] is a self-report personality scale to assess individual differences in propensity to disgust. There are 25 disgust elicitor items loading on one of the three factors (core, animal reminder, and contamination-based disgust) and two catch questions (items 12 and 16) to identify those respondents that are not paying attention to the task or do not take it seriously. Each item is rated by the participant on a 5-point Likert scale from 0 (“Strongly disagree/Not disgusting at all“) to 4 (“Strongly agree/Extremely disgusting”). The total score (ranging from 0 to 100) is calculated by summing scores on all the 25 disgust elicitor items but three (items 1, 6, 10) that are reverse scored. Similarly, subscale scores may be calculated. All the participants that do not give valid answers to the catch questions should be dropped. The DS-R demonstrates acceptable Cronbach’s alpha estimates for the overall internal consistency (0.84) and the three subscales [core disgust: 0.74; animal reminder disgust: 0.78; contamination-based disgust: 0.61: (47); see also (48, 49)].

### 2.3. Behavioral approach test (BAT)

**Figure 1:**
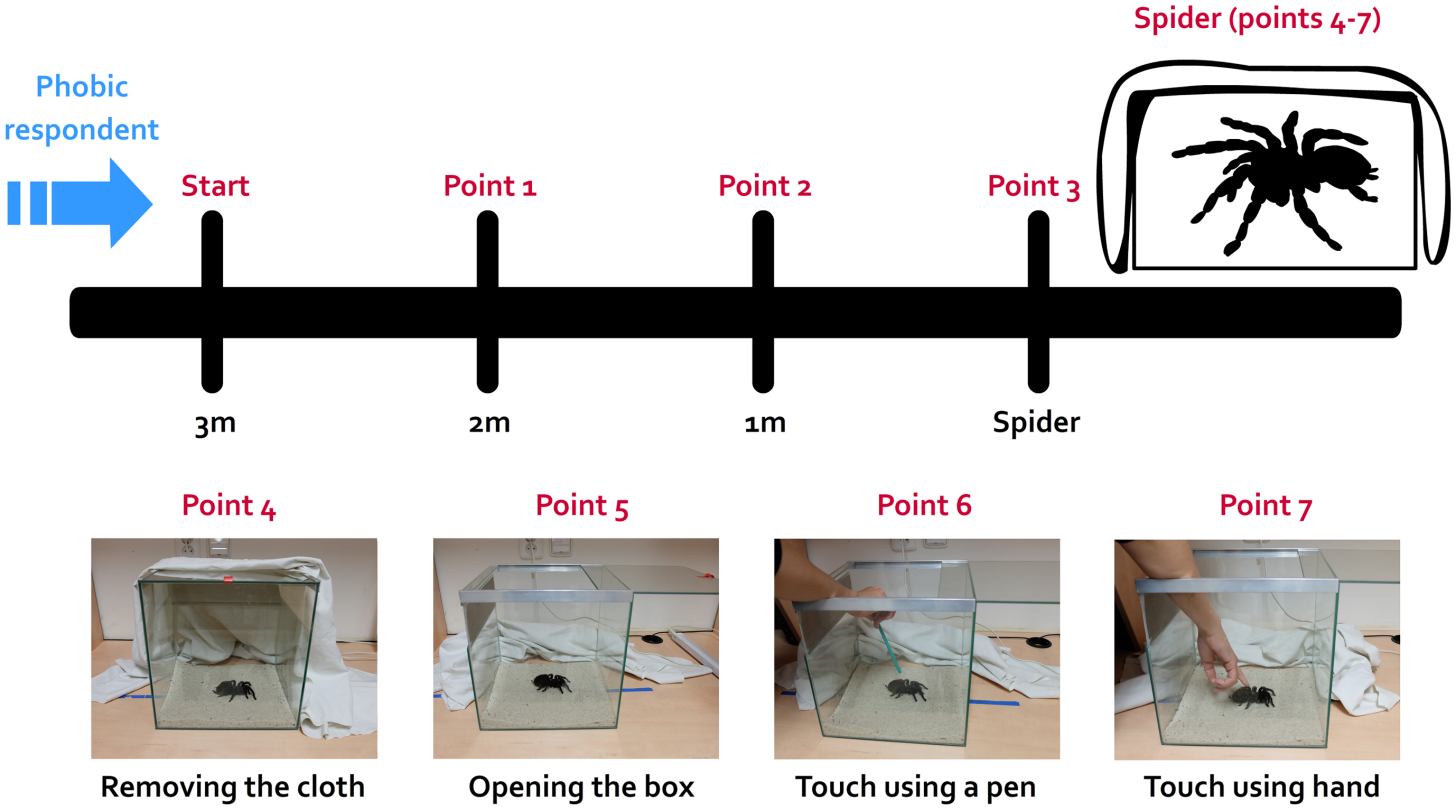
Schematic design of the behavioral approach test (BAT). The BAT was performed in a separate room, where a completely covered terrarium with a harmless brown-colored spider (Mexican red-rump tarantula *Tliltocatl vagans*) was placed. The respondent was asked to enter to room and approach the spider in a stepwise manner as close as possible (they could end the task at any point), even touch it with their finger in the last step. The scheme represents each step during the test (completing each step was honored by one point, 7 points total): moving closer to the terrarium (points 1 to 3), removing the cloth and looking at the spider (4), opening the terrarium (5), touching the spider with a pencil (6) and ultimately touching it with a finger (7).

This task measures fear and behavioral avoidance in response to live spiders. An individual of a harmless species (a big spider commonly known as the Mexican red-rump tarantula; *Tliltocatl* (ex *Brachypelma*) *vagans*) that was accustomed to regular manipulation (handling) was placed in a terrarium covered with a white cloth at one end of a room. The respondents were asked to enter the room and approach the animal as close as possible, ultimately touching it. The BAT was constructed in a stepwise manner, each step corresponding to one scale point (7 points total). The subjects were first instructed to move 1 m toward the spider (BAT score = 1), then another meter (BAT = 2), then to approach the terrarium (BAT = 3), then to remove the cloth and look at the animal (BAT = 4), to open the terrarium (BAT = 5), to touch the animal with a pencil (BAT = 6), and finally to touch the animal with a finger (BAT = 7); the BAT procedure is depicted in Figure 1. The participants were instructed they can end this task at any point. The BAT score thus corresponded to how many steps the respondent was able to complete.

Phobics completed on average 4.6 steps out of 7 in the BAT compared to controls with an average score of 6.8. Half of the phobic subjects were only able to uncover the closed terrarium and look at the spider, while the other half were able to open the terrarium and touch the spider with a pen or even their hand (*n* = 2); see Results. Most of the control subjects touched the spider with their hand. In general, this experiment can distinguish between true spider phobics and false positives with high SPQ scores, yet not phobics.

### 2.4. Fear of snakes and other variables from a larger experimental study

The SFI variable is computed from data gained during a larger experimental study. The main focus of that study was to compare fear of spiders in spider-phobic respondents during two different priming conditions: fear and disgust. The full details will be described elsewhere and are not important for the current study. However, because the experiment included pictures of snakes as stimuli depicting “generalized fear” (i.e., non-phobic), all respondents in the study were also selected based on their SNAQ scores (no respondent in the study was allowed to have a SNAQ score higher than 17, and the mean score was 2.71). Moreover, the respondents scored all the stimuli seen in the larger experiment on a 7-point scale according to fear, as described below.

### 2.5. Picture stimuli

There were six categories of picture stimuli, all presented using a block-type design in magnetic resonance. One category consisted of 40 unique and 40 horizontally transformed pictures, together consisting of 80 pictures per category. The categories were: big spiders (including various pictures of the Mexican red-rump tarantula; further referred to as tarantula), small spiders (including the daddy-long-legs spider, *Pholcus phalangioides*), beetles (including a female Asiatic rhinoceros beetle, *Oryctes nasicornis*), leaves, venomous viperid snakes, and lizards.

**Figure 2:**
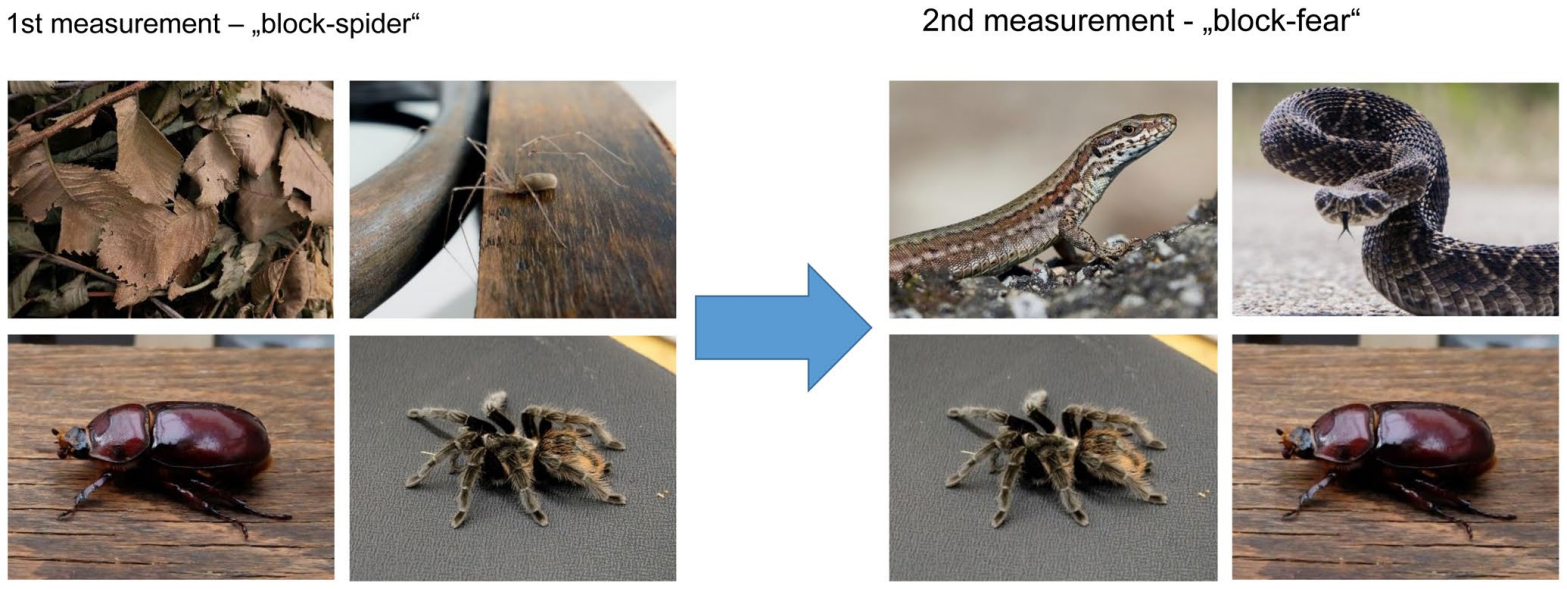
Visualized scheme of the experimental fMRI procedure. The block design experiment was performed in two measurements (further called “spider” and “fear”), each separated by a short time during which the subject was resting inside the tunnel. Each measurement consisted of pictures of one of four different conditions: Mexican red-rump tarantula (*Tliltocatl vagans*), daddy-long-legs spider (*Pholcus phalangioides*), beetle (*Oryctes nasicornis* female), and leaves for the “spider”; and spider, beetle, fear (venomous snakes) and fear-control (lizards) for the “fear” block.

The part of the experiment during which the SFI was measured consisted of a presentation of the stimuli in two sessions: the “Spider” session (with the two spider categories and leaves and beetles as control categories) and the “fear” session (consisting of the tarantulas, beetles, snakes, and lizards, see Figure 2). Each category was presented in blocks of 10 pictures, pseudorandomly so that each category follows one another the same number of times. Each picture within a block was presented for 1.5 s, which corresponded with the data acquisition time *T* = 1.5 s. The presentation of the first session lasted 8 min, after which there was a small (about 10 min) break for the measurement of morphological data during which the respondents could rest inside the magnetic resonance. After then, the second session followed.

**Figure 3:**
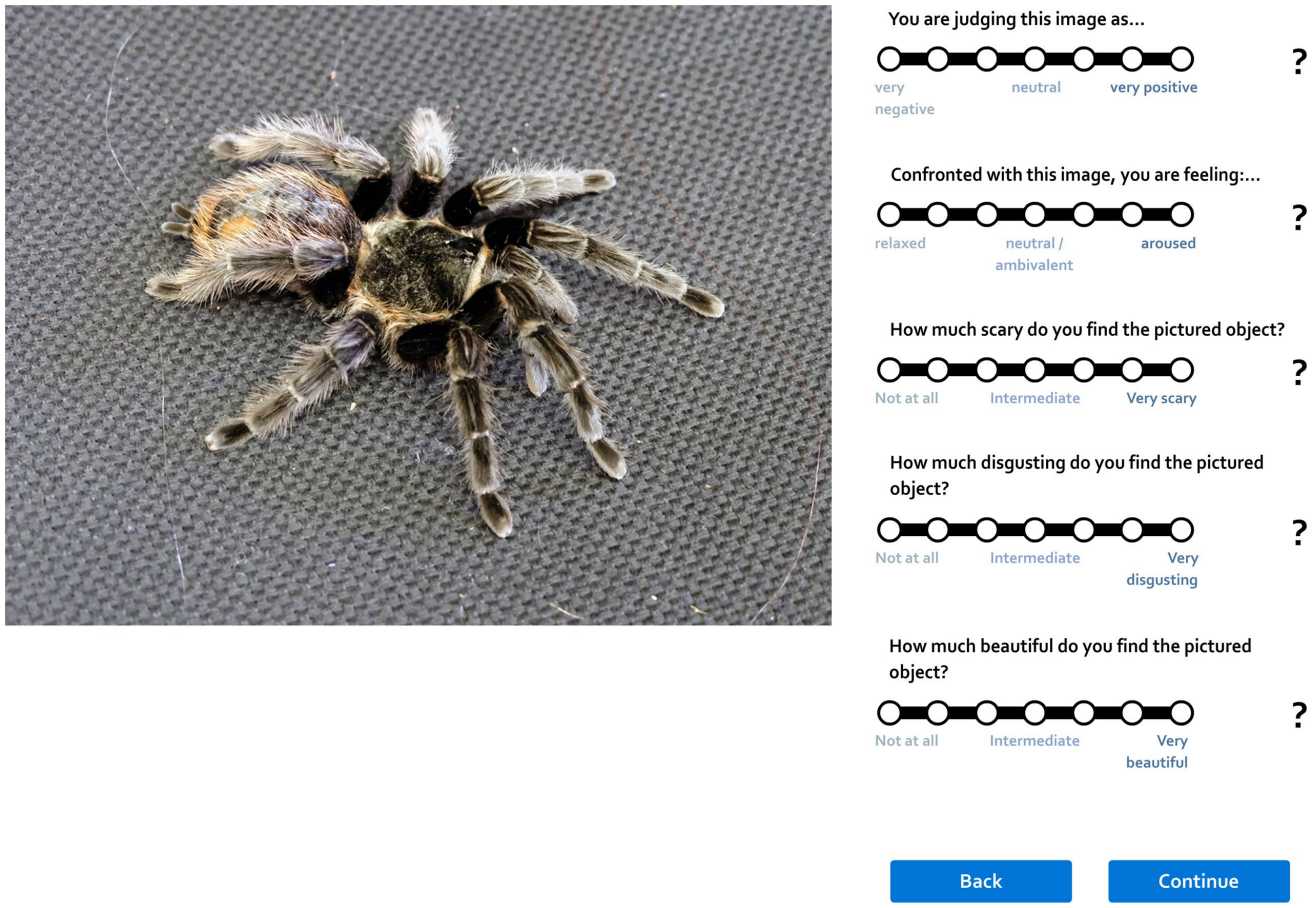
Preview of the web application that allows for subjective scoring of pictures. Please note that this study was a small part of a complex experiment; thus, while each stimulus was scored on various dimensions, only the fear scores were reported here. Another paper reporting the full results of the complex study is in preparation.

When the MR sessions ended, the respondents were seated in front of a computer and asked to sign up/login into our application at www.krasazvirat.cz which allows for rating stimuli or filling out various questionnaires. After logging in, they started to rate the 40 unique pictures per category as seen during the fMRI presentation according to their perceived fear on a Likert scale ranging from 1 (no fear) to 7 (strong fear; see Figure 3). The pictures appeared on the computer screen in an order randomized for each respondent.

### 2.6. fMRI data acquisition, preprocessing, and analysis

Functional data were collected on a 3 T MR scanner Siemens Prisma (Siemens Medical Systems, Erlangen, Germany) equipped with a 64-channel head coil. The stimuli were rear-projected onto a mirror mounted on the head coil. Functional images (using the BOLD method) were acquired using a gradient-echo echo-planar imaging sequence (GE-EPI) covering the whole brain with 52 slices and a voxel size of 3 × 3 × 3 mm^3^. This functional sequence had the following parameters: Field of view (FOV) = 216 × 216 mm, repetition time/time to echo (TR/TE) = 1.500/30 ms, a flip angle of 52°, and multiband factor 2. During the functional measurement, a total of 320 scans of brain volumes were acquired.

First, bias field correction was performed to correct for inhomogeneities caused by the 64-channel head coil, followed by a spatial realignment of all images to correct for possible head motion in the scanner, a slice-timing correction to compensate for the delay in the acquisition times of the axial slices, normalization of the images into the standard MNI space, and spatial smoothing with Gaussian kernel of 8 mm full width at half maximum (FWHM).

**Figure 4:**
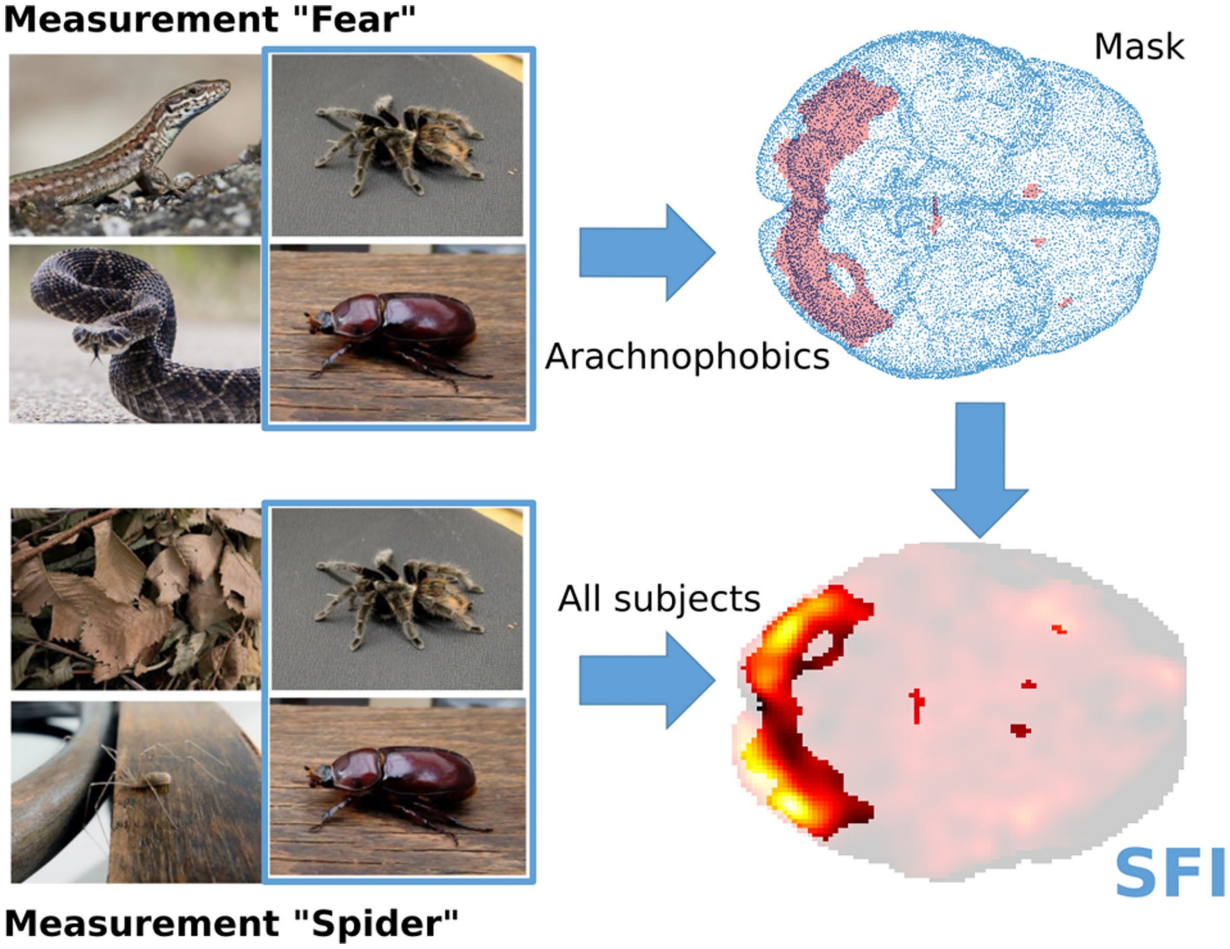
Preview of the idea of Spider fear index (SFI) computation. The mask, i.e., the set of voxels reacting to the spider compared to the beetle, was computed from the Arachnophobic group of subjects during the measurement „Fear“. Then, SFI was computed from the measurement “Spider” for every subject as the average activation strength, restricted to the previously defined mask, using the same contrast “spider>beetle.”

The experiment consisted of three measurements (block-Fear, block-Disgust, block-Spider) with 4 types of stimuli each. For our analysis, we used the contrast Spider>Beetle from the measurement Fear and Spider (see Figure 4 for a simplified scheme of the experimental design). On the subject-level analysis, the generalized linear model was used, and the activation increases were calculated via a one-sample t-test. The obtained activation maps were later used for the Spider Fear Index (SFI) computation.

Although 30 phobic individuals underwent the fMRI measurement, one subject had to be excluded from further analyses due to the low quality of acquired fMRI data.

### 2.7. Spider fear index

To study the specific neurophysiological reaction to spiders, we propose the Spider Fear Index (SFI). This measure is an outcome of a larger experimental study (in prep.) of neural activation of spider-phobic versus control respondents. In this study, “spider “always refers to the pictures of a tarantula included as the main focus of the study. The SFI is computed from the “block-spider” fMRI measurement, where blocks with both tarantulas and long-legs spiders and beetles are first presented. To define the SFI, we aimed to calculate a value representing the average brain activity in a spider-specific region of interest (ROI) during spider observation. The mentioned specific ROI is defined independently of the studied fMRI measurement. Thus, we define SFI as an average increase in brain activation while observing spiders, where beetles were taken as a control condition, computed in a predefined ROI.

**Figure 5:**
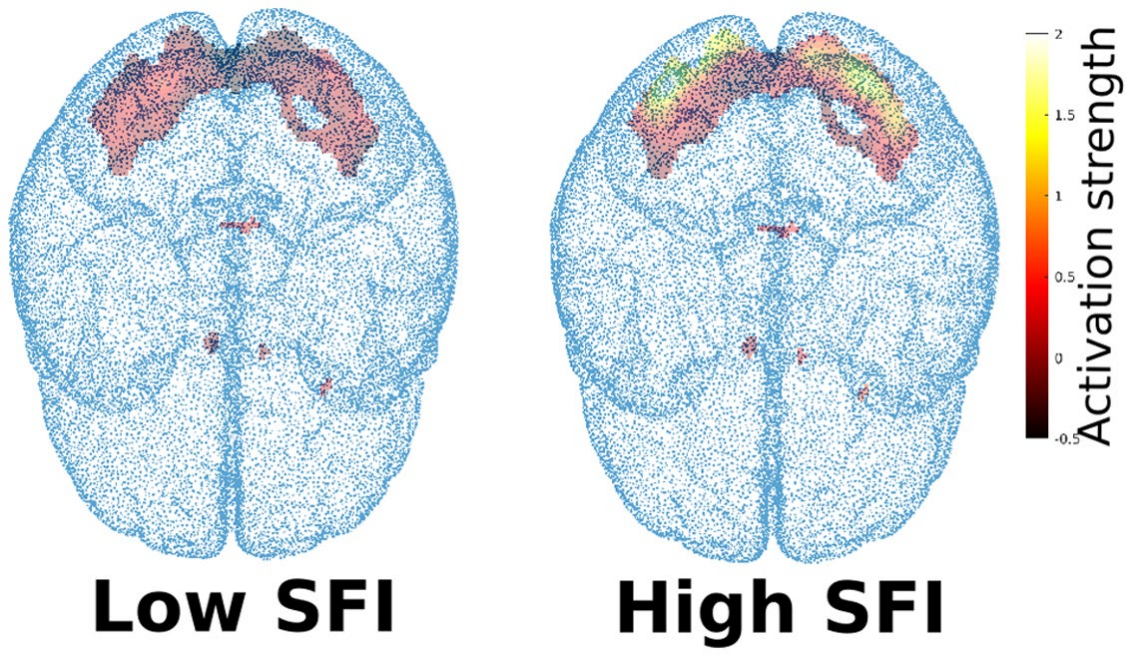
Example of higher and lower Spider fear index (SFI). Activation strength is given by the t-statistics, obtained during the subject-level statistical analysis of the fMRI data.

More technically speaking, the ROI was computed on the group of arachnophobic respondents using the measurement “block-fear.” It was defined as a set of significant voxels for the contrast Spider>Beetle (group level analysis, significance level 0.05, FWE correction). Then, for all studied subjects, SFI was defined as an average value of the t-statistic in predefined ROI, computed from the measurement “block-spider” (see Figure 3). This means that SFI was computed as an average first-level map for the condition Spider>Beetle, masked to the specific ROI. In our sample, the SFI gained values from −0.0173 to 0.118, for example, see Figure 5.

We then treated this variable as the “neurophysiological effect of spiders,” or implicit measurement of spider fear, unique to each respondent; and we examined its contribution to the respondents’ fear scores ratings of the stimuli seen during the fMRI measurement. The effect was examined using the redundancy analysis [RDA; implemented in the R package vegan; (50)]. RDA is a multivariate direct gradient method. It extracts and summarizes the variation in a set of response variables (subjective evaluation of fear elicited by pictures) that can be explained by a set of explanatory variables (SFI, questionnaires, behavioral response, etc., see Statistical analysis for the details). This analysis permits to plot both responses and explanatory variables to a space defined by the extracted gradients and enables to detect redundancy (i.e., shared variability) between sets of response and explanatory variables.

### 2.8. Statistical analyses

The general aim of this study was to examine relationships between various indexes, scores, and ratings commonly used to characterize spider fear propensity. Firstly, we utilized the method of redundancy analysis (RDA). It extracts the variation in a set of response variables that can be explained by a set of explanatory variables, allowing us to examine multivariate relationships between multiple response and explanatory variables at the same time (51). We examined the contribution of respondents’ age, grouping (phobic or control), SFI, SPQ, SNAQ, DSR, and BAT scores to the respondents’ subjective fear ratings of the stimuli presented during the fMRI measurement. The statistical significance of the gradients was confirmed by permutation tests.

Next, linear models (LM) were used to analyze how much variability of the SFI can be explained by subjectively measured fear scores. The full model included BAT, SPQ, SNAQ, and DS-R scores, tarantula, daddy-long-legs spider, leaves, beetle, lizard, and snake fear scores, as well as participants’ grouping and age. The best model was chosen based on Akaike Information Criterion. We also performed the Discriminant Function Analysis (DFA) to investigate which variables would successfully divide the respondents into the two groups of phobics and controls. Lastly, we checked the Spearman correlation between SFI and BAT and SPQ scores to access whether the implicit component of fear (as expressed by SFI) would be contained in the latter two variables, as well. DFA was performed in the software Statistica (52), and the remaining analyses in R (53), using packages vegan (50) and MASS (54). The analysis of the fMRI data was done by MATLAB v. 2020 via SPM12, which was used for preprocessing and statistical analysis. The Adult MNI-ICBM152 head model (55) was used for visualization.

### 2.9. Ethical note

All procedures performed in this study were carried out following the ethical standards of the appropriate institutional research committee (the Ethic Commission of National Institute of Mental Health, approval no. 117/18, granted on 28 March 2018), and with the 1964 Helsinki Declaration and its later amendments or comparable ethical standards. Written informed consent was obtained from all participants included in the study.

## 3. Results

### 3.1. RDA

**Figure 6:**
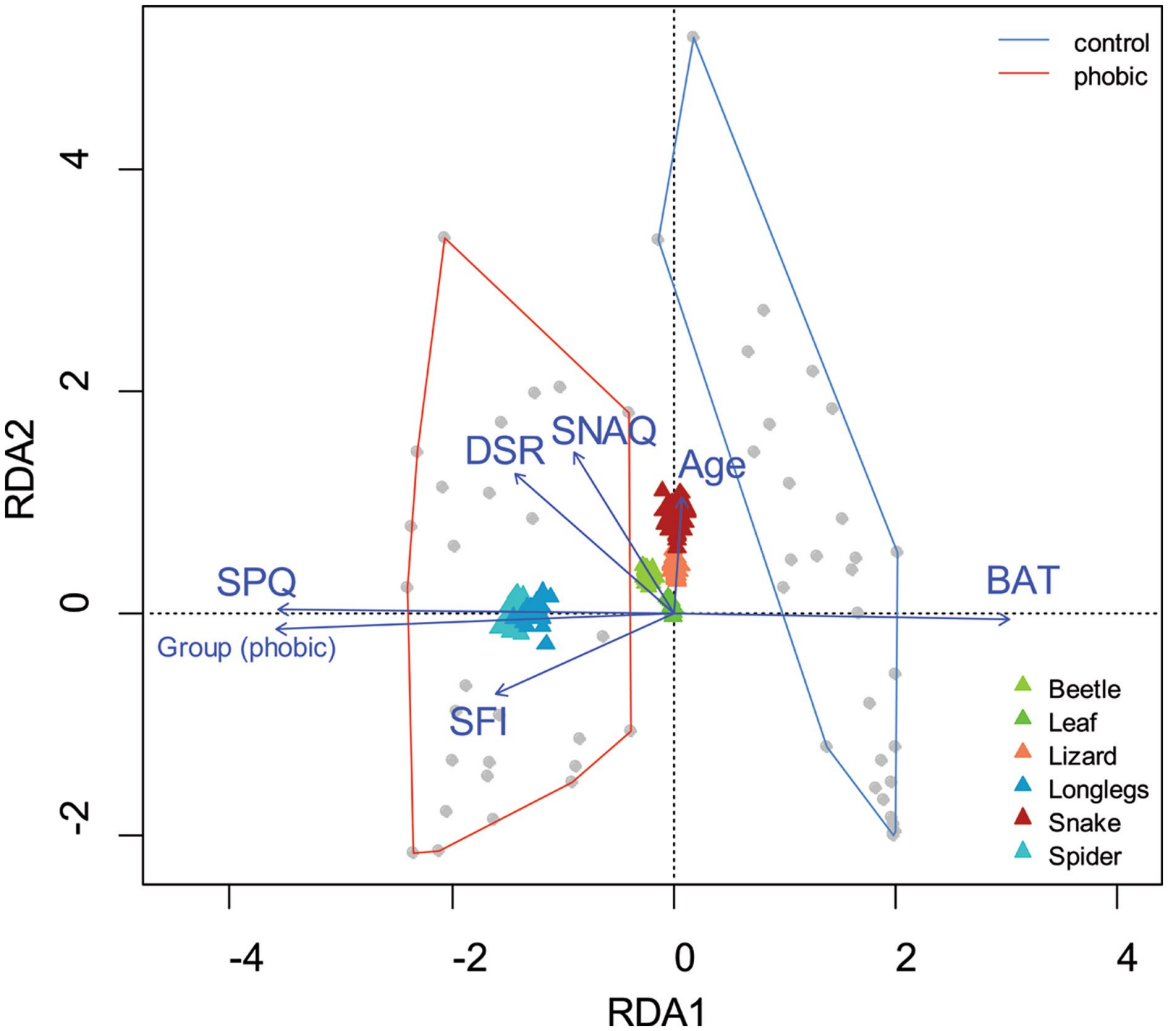
Visualization of the redundancy analysis (RDA) of fear ratings of the visual stimuli. Redundancy analysis (RDA) of the respondents’ age, experimental group status (phobic, control), score on the Snake Questionnaire (SNAQ), Spider Questionnaire (SPQ), Disgust Scale - Revised (DSR), Behavioral approach test (BAT), and Spider fear index (SFI) as explanatory variables and fear ratings of the tested picture stimuli as response variables. The grey dots represent the respondents, while the colorful triangles represent the fear rating of the picture stimuli. Blue arrows signify the direction of the explanatory variables’ effect; the longer the arrow, the stronger the effect. The RDA reveals a clear segregation of the phobic respondents from the controls (delineated manually by the red and blue lines, respectively, based on the minimum convex polygon method). The first axis is fed mainly by the respondents’ subjective fear of spiders, while the second axis corresponds to the fear of snakes (as revealed by the reduced model). The model explained 61.95% of the full variability.

We examined the contribution of the SFI to the respondents’ subjective fear ratings of the stimuli as seen during the fMRI measurement. The full model (*n* = 61) included the grouping of the respondents (phobic/control), SFI, SPQ, SNAQ, DSR, and BAT scores, and age. The RDA model of fear ratings has generated seven constrained axes, which explained 61.95% of the full variability. The sequential “Type I” test (n permutations = 2,000) revealed that only the SFI [*F*(1,53) = 16.283, *p* < 0.001], SNAQ [*F*(1,53) = 9.956, *p* < 0.001], DS-R [*F*(1,53) = 7.424, *p* = 0.003] and SPQ scores [*F*(1,52) = 46.978, *p* < 0.001] had a significant effect (see Figure 6).

### 3.2. LM and discriminant function analysis

**Figure 7:**
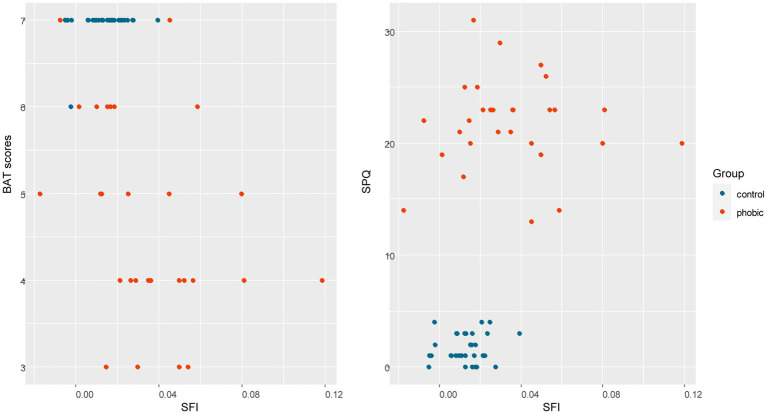
A plot of the SFI and SPQ/BAT scores. The phobic group of respondents was defined by a clinical psychologist based on an interview with each respondent. These plots show that SPQ X SFI separates two distinct groups as was initially predicted, while BAT X SFI contains a few intersections of both groups. In both plots, higher variability in SFI in the phobics group is apparent.

To analyze how much variability of the SFI can be explained by subjectively measured fear scores, we performed an LM analysis in R Statistics. The full model included BAT scores, SPQ, SNAQ, and DS-R scores, tarantula, daddy-long-legs spider, leaves, beetle, lizard, and snake FS, and group and age, but only the BAT scores [*F*(1,48) = 26.517, *p* < 0.001] and snake FS [*F*(1,48) = 8.9810, *p* = 0.004] were significant. The reduced model (based on Akaike) explained 45.99% of total variability and included BAT scores, tarantula, daddy-long-legs spider, lizard, and snake FS, and age, but again only the BAT scores [*F*(1,54) = 28.387, *p* < 0.001] and snakes FS [*F*(1,54) = 12.667, *p* < 0.001] remained significant (for a plot between SFI and BAT/SPQ, see Figure 7).

We performed the Discriminant Function Analysis to examine which variables would successfully discriminate the respondents into the two groups of phobics and controls. The strongest discrimination variable was the tarantula FS (*F* = 146.76, *p* > 0.001), followed by the snakes FS (*F* = 45.78, *p* > 0.001), daddy-long-legs spider FS (*F* = 12.55, *p* > 0.001), and SNAQ scores (*F* = 10.14, *p* = 0.002). The discrimination resulted in 100% correct assignment to the phobic/control groups (see Table 1).

**Table 1:** Results of the discriminant function analysis.

	Percent	Phobic	Control
Phobic	100	29	0
Control	100	0	32
Total	100	29	32

The discrimination, based on the SNAQ scores and the tarantula, long-legs spider, and snakes fear scores was 100% successful when discriminating into the phobic and control groups.

### 3.3. Correlation

Finally, we looked at the correlations that were to reveal whether there is an implicit component of fear present in the approach behavior toward the spider and the self-reported scoring of fear perceived from spider pictures. SPQ was strongly correlated with both BAT (rs = −0.83, *p* < 0.001) and fear scores (rs = 0.815461, *p* < 0.001). The SFI was also significantly correlated with both BAT (Spearman rs = −0.51654, p < 0.001) and fear scores (rs = 0.44, *p* < 0.001), but only moderately. Next, we checked whether there is a relationship within each other of these variables, and we also found significant correlations: SFI correlated with SPQ moderately (rs = 0.4311439, *p* < 0.001), BAT correlated with the fear scores strongly (rs = −0.8369, *p* < 0.001), see Figure 7. Note, that all these above-mentioned correlations might be a consequence of the separation of our respondents into contrasting phobic and control groups and the statistics should therefore be treated with caution.

## 4. Discussion

In this study, we aimed to compare the behavioral approach toward a live spider and fear scores assigned to spider pictures by phobic and control respondents with variables that correspond to the measurement of implicit and explicit compounds of spider fear. The implicit compound was measured as a neurophysiological brain activation: the fMRI BOLD signal (and calculated as SFI).

A typical way to analyze the neuronal response in fMRI studies linked to specific phobia consists of the block- or event-design paradigm of the experiment and hypothesis-driven ROI analysis or the whole-brain voxel-wise analysis. The typical outcome of these kinds of approaches utilizes the single subjects’ first-level analysis statistic maps to compute the group-level result, whose comparison with other variables, such as questionnaire scores, might not be straightforward. Hypotheses formulation might also be tricky. Moreover, defining the ROI of phobia-specific areas can present quite a challenge. Several reviews [e.g., (56–58)] provide an overview of studies of specific phobias with fMRI. Despite those reviews that found a group of similar brain areas related to brain responses to phobic stimuli, several other areas were also found to be associated with phobias (59), showing the grasp of such an area may sometimes prove to be hard.

Our approach addresses both mentioned issues. We define the data-based ROI instead of constructing it based on the literature, which is heterogeneous both in the design study (measurement parameters) and results (56, 57, 59–62). Then, the average of the first-level statistical map is computed per subject, which is an easy-computable, relatively interpretable, one-dimensional feature, describing the intensity of activation rather than its exact location. Such a variable can be then easily used in analyses with other measurements of spider fear that describe various levels of fear experience.

The correlations showed that BAT and spider fear scores were more closely correlated with the direct measurement of subjective fear using the SPQ, which supports the hypothesis that the main component is the controlled, explicit fear reaction. However, both variables were also significantly correlated with SFI. The correlation was lower but still reflects a moderate significant influence of the automatic, implicit reaction of fear in BAT and during subjective scoring of visual spider stimuli. This finding is very important as it highlights the potential of visual stimuli scoring as a cheap, widely available yet efficient measurement of phobic fear. Moreover, our results showed that spider-phobic respondents do not discriminate much among various spider morphotypes: both the large tarantulas and small, brittle daddy-long-legs spiders showed to be good discriminants of the spider-phobic group of respondents. Thus, it is possible that pictures of most spiders can trigger the fearful response of arachnophobic subjects (29), which allows for even easier and more widely applicable research. The inclusion of this method in diagnostics of phobic respondents or patients can improve the detection as this measurement probably includes not only explicit but also implicit fear reactions toward spiders. Moreover, our results showed that BAT should not be considered as strictly or mainly explicit testing, which should be reflected in future studies (38).

One of the few similar studies was performed in Germany by Mühlberger et al. (33). Like us, they measured various levels of fear, but their variables slightly differed from ours: their focus was a behavioral approach conducted in virtual reality (VR-BAT), which they compared to psychometric measures (SPQ, SBQ, and others), physiological measures (heart rate and skin conductance), and subjective fear ratings (Subjective Unit of Discomfort during the VR-BAT). In contrast with our results, the authors found discordance between the fear reports and the physiological fear responses; however, they also found a small yet significant correlation between the approach and the subjective ratings with the SPQ scores, and a fairly high correlation of the approach with the subjective fear scores (55 and 62% in two different VR-BAT trials). This discordance with our results may be caused by the fact that they used the Subjective Unit of Discomfort as an output variable of the behavioral approach. Instead of measuring the speed or distance from the spider, which itself can include the automatic fear component, the respondents were to subjectively score the level of anxiety they experienced during the VR-BAT, which might have favored the controlled response during the test.

Our results also revealed that our measurements of explicit and implicit components of fear were moderately correlated. This was revealed also during the RDA analysis, which showed that SPQ and SFI both pointed out the same direction (on the X-axis), although the effect of SFI also contributed to the y-axis described by the common effect of snake fear, age, and DS-R. Thus, in our study, although the effect of both implicit (BAT and SFI) and explicit fear reactions (SPQ) was found, these components were not affecting the behavior independently. However, the RDA results showed clearly that the BAT lies at the same axis as the SPQ scores, and the fear scores are mainly formed by this first axis, reflecting the controlled fear reaction.

The role of disgust propensity [individual tendency to experience disgust, (48)] measured by the DS-R contributes to the first multivariate axis of RDA, i.e., it is positively correlated with individual sensitivity to fear of spiders (SPQ scores) and negatively with BAT scores (higher scores in the test represent lower fear of spiders, see Figure 1). In the same direction as the DS-R also goes the spider fear index (SFI) and the SNAQ measuring individual sensitivity to fear of snakes, see Figure 6. However, the influence of disgust emotion on subjective fear evaluation of the picture stimuli of spiders, snakes, and lizards and its relationship to the other measured parameters is complicated. The contribution of disgust propensity measured by the core disgust and animal reminder subscale of the DS-R to fear of spiders measured by the SPQ was previously independently confirmed in our study that included nearly nine hundred respondents (17). Our previous and current results are both in line with other studies showing that disgust propensity, but not disgust sensitivity (the degree to which an individual is distressed by their experience of disgust) is related to spider fear (63, 64). However, the DS-R scores correlate with other parameters as well.

Interestingly, our results show that the disgust propensity also contributed to the second axis and might be correlated with the brain activation (SFI) as well as the individual sensitivity to fear of snakes (SNAQ, see the paragraph below). Unfortunately, the generalized linear model did not confirm the direct contribution of disgust propensity to the brain activation represented by the SFI in our set of sixty-one respondents. The relationship between brain activation when seeing a spider picture and experiencing different levels of fear and disgust (affected by the individual disgust propensity) must be explored further.

According to the glm analysis, the SFI obtained from brain activations elicited by spider stimuli was mostly explained by the BAT scores, but also by snake fear scores. It is interesting because snake phobics were not included in the study. The sensitivity to the fear of spiders and sensitivity to the fear of snakes may be slightly correlated (see Figure 6), as was also shown previously in the study working with a large dataset (17). The connection between the subjective emotional evaluation of spiders and snake pictures as well as individual sensitivity to fear of snakes was important also for the correct identification of phobic respondents. The discrimination analysis showed 100% discrimination of the phobic respondents based on the tarantula, snakes, and daddy-long-legs spider scores and the individual sensitivity to the fear of snakes measured by SNAQ. Still, the spider scores proved a very good discriminating factor itself and further underlined the potential value of visual stimuli in the assessment of spider fear.

In the current study, we compared two groups of participants, spider phobics and healthy subjects with low fear of spiders. In this case, individual subjective emotional evaluation of picture stimuli that combines an implicit as well as explicit component of emotional evaluation separates these two groups of respondents. The level of avoidance behavior (BAT score) confirms this division. The intensity of brain activation during watching the spider stimuli (SFI) corresponded with the above-mentioned factors, but its statistical significance was not confirmed by a statistical test. We can speculate that higher variability of individual brain activation (SFI) during the fMRI task among the phobic respondents compared to the control group might have caused this effect. Approximately half of the phobic respondents showed a similar intensity of brain activity as the control group, but the other half had significantly higher brain activations, see Figure 7. This means that only some phobic individuals show significantly increased brain activation when exposed to a phobic stimulus in fMRI, although this feature is then considered one of the core characteristics presented in most phobic patients (65, 66).

Many physiological studies showed an increased response in spider-phobic individuals, although there still might be a substantial level of individual variability (67) similar to our findings. However, in the current study, we did not investigate individual increased or decreased activity in certain areas responsible for the proper regulation of experienced emotions, which may also play a crucial role, especially in the treatment of phobias, e.g., performing cognitive behavioral therapy (62). Not only fMRI studies but also ERP studies focusing on the role of early and late attention can help us understand the relationship between implicit and explicit emotional experience in phobics. Soares et al. (68) showed in healthy participants that early attention (associated most probably with implicit emotional perception) is weaker in spiders, but it is then compensated by an increase of parameters corresponding with late attention, which may be more related to explicit processing of the spider stimulus. For a better understanding of how implicitly and explicitly perceived emotions are involved in specific phobias, it should be interesting to compare the subjective evaluation of multiple picture stimuli, avoidance behavior (BAT), and SFI index extracted from fMRI with the above-mentioned component of early and late attention in spider phobics in the future studies.

## 5. Conclusion

We confirmed that the physiological measurement (based on fMRI) of spider-phobic respondents, which represents an implicit fear reaction to spiders, corresponds to the subjective scoring of spider pictures and BAT results. While not surprising, this result is very important because it confirms the credibility of scientific work that was built on subjective measurements. Moreover, the results show that future studies with phobic people dealing with the intensity of perceived emotion can be, without fear, performed without expensive equipment and time-consuming methods of neuroimaging measurement. This study confirms that psychologists and psychiatrists can easily and reliably determine the patient’s condition based on questionnaires and BAT, especially when they include visual phobic stimuli. During an examination, simple scoring of a few static photos of spiders may reliably reveal the patients’ phobic fear of spiders without the need for expensive or complex tools. This attitude is valid especially when we ask about the intensity of perceived emotion, not about a particular part of the brain or brain network participating in emotional processing and regulation. SFI might however be a good measure reflecting individual sensitivity to implicitly perceived emotions in phobic respondents.

## Data availability statement

The original contributions presented in the study are included in the article/supplementary material, further inquiries can be directed to the corresponding author/s.

## Ethics statement

The studies involving human participants were reviewed and approved by the Ethic Commission of National Institute of Mental Health. The patients/participants provided their written informed consent to participate in this study.

## Author contributions

EL and DF designed the study. MJ, ŠP, KS, IŠ, and JP collected the data. IŠ did curation of the data. JH, AP, and DT did curation of the fMRI data. DF performed statistical analyses. AP, DT, and JH performed statistical analyses of the fMRI data. MJ and ŠP created the photographic stimuli. SR, EL, and JP wrote the manuscript. JP administrated the project. All authors contributed to the article and approved the submitted version.

## Funding

This project has been supported by the Czech Scientific Foundation (GAČR), project No. 19-07164S, awarded to EL, https://www.gacr.cz/en/.

## Conflict of interest

The authors declare that the research was conducted in the absence of any commercial or financial relationships that could be construed as a potential conflict of interest.

## Publisher’s note

All claims expressed in this article are solely those of the authors and do not necessarily represent those of their affiliated organizations, or those of the publisher, the editors and the reviewers. Any product that may be evaluated in this article, or claim that may be made by its manufacturer, is not guaranteed or endorsed by the publisher.
